# Dynamic hip screw fixation of subtrochanteric femoral fractures

**DOI:** 10.1007/s00590-021-02895-4

**Published:** 2021-02-16

**Authors:** Zaki Arshad, Azeem Thahir, Jaikirty Rawal, Peter D. Hull, Andrew D. Carrothers, Matija Krkovic, Daud T. S. Chou

**Affiliations:** 1 University of Cambridge School of Clinical Medicine, Box 111 Cambridge Biomedical Campus, Cambridge, CB2 0SP UK; 2 Cambridge Orthopaedic Trauma Unit, Addenbrooke’s Hospital, Cambridge University Hospitals NHS Foundation Trust, Cambridge, CB2 0QQ UK

**Keywords:** Hip fractures, Internal fixation, Subtrochanteric fractures, Hip screws, Intramedullary nails

## Abstract

**Introduction:**

A subtrochanteric proximal femur fracture occurs in the 5 cm of bone immediately distal to the lesser trochanter. UK national guidelines advise that adults with subtrochanteric fractures should be treated with an intramedullary nail (IMN). This study aims to compare peri-operative outcome measures of patients with subtrochanteric fractures treated with either an IMN or a dynamic hip screw (DHS) construct.

**Materials and Methods:**

We retrospectively reviewed subtrochanteric fractures presenting at our institution over 4.5 years (October 2014–May 2019), classifying them into two treatment groups; IMN and DHS. These groups were compared on outcome measures including surgical time, blood loss, radiation dose area product (DAP), length of stay, re-operation rate and mortality.

**Results:**

During the time period studied, 86 patients presented with a subtrochanteric fracture of the femur; with 74 patients (86%) receiving an IMN and 12 (14%) receiving a DHS. The comparative outcome measures reaching statistical significance were blood loss and radiation DAP. The DHS group showed a significantly lower mean blood loss of 776 ml compared to 1029 ml in the IMN group. Also, the DHS group showed a significantly lower mean DAP of 150.30 mGy cm^2^ compared to 288.86 mGy cm^2^ in the IMN group.

**Conclusion:**

Although UK national guidelines recommend treating all subtrochanteric fractures with an IMN; the outcome measures assessed in our study did not show use of an IMN to be superior to a DHS. The DHS group showed a lower estimated blood loss and a reduced DAP. This, along with the reduced financial cost associated with a DHS, may support the use of DHS over IMN for certain subtrochanteric fractures of the femur. There may not be a single favourable implant for the treatment of subtrochanteric fractures as a whole; instead different subtypes of fracture may be amenable to a number of fixation devices. Choice of implant should be determined locally and based on existing and future clinical and health economic research.

## Introduction

Hip fractures are the most common reason for admission to an orthopaedic ward in the United Kingdom (UK), with over 86,000 occurring every year [1]. The majority of hip fractures are due to ‘fragility’ fractures in older people with osteoporosis or osteopenia presenting after a fall [2]. This is reflected in the average age of people presenting with hip fractures being 77 [2]. The population of the UK and several other countries is becoming increasingly older, which is only likely to exacerbate the prevalence of such fractures [3]. This, together with the fact that the combined cost of medical and social care for all hip fracture presentations in the UK amounts to around £1 billion means that the appropriate management of such fractures, is of crucial importance [2].

Hip fractures are often grouped into intracapsular or extracapsular fractures. Our study will focus on the extracapsular group and more specifically on the subtrochanteric fractures. These fractures affect the region of the proximal femur within five centimetres distal to the lesser trochanter. They account for around 5% of proximal femur fractures and previous studies report a bi-modal age distribution consisting of young adults involved in high energy traumas and older patients with osteoporosis [4, 5].

The UK national guidelines currently state that all subtrochanteric fractures should be treated using an intramedullary nail (IMN) [2]. This is due to concerns with reported high rates of femoral medial displacement, non-union and subsequent re-operation in those with unstable subtrochanteric fractures treated using a dynamic/sliding hip screw (DHS). However, the DHS remains a method for fixation in subtrochanteric fractures, with several surgeons continuing to use it due to its ability to provide compression along the femoral neck as well as load sharing between the bone and implant [6–8]. UK national guidelines, however, recommend that such extramedullary implants only be used in the treatment of adults with trochanteric fractures above and including the lesser trochanter [2]. This study aims to compare some peri-operative outcome measures of a group of patients with subtrochanteric fractures treated with either an IMN or a DHS construct.

## Materials and methods

Using the National Hip Fracture Database, we retrospectively reviewed all patients presenting with a hip fracture to our major trauma centre over a 4.5-year period (October 2014–May 2019). We selected the patients presenting with a subtrochanteric fracture specifically and used the electronic patient record system to gather relevant data about each patient. Patients were first grouped based on the treatment received—IMN or DHS. Demographic data were collected including patient age, gender, laterality, Body mass index (BMI) and American Society of Anaesthesiologists (ASA) physical status classification. All fractures were classified using the Seinsheimer classification system [9]. This system classifies subtrochanteric fractures into one of 8 possible subtypes and has been used in a number of similar previous studies [10–14]. Fracture stability was defined according to wang et al., which considers Seinsheimer type i and ii fractures as stable and type iii, iv and v fractures as unstable [15].

All operations were performed at a Major Trauma Centre by either a consultant orthopaedic surgeon or an orthopaedic trainee supervised by a consultant. Due to the retrospective nature of this study, we were unable to define a -priori criteria outlining specific situations in which a DHS or IMN should be used. Instead implant choice was determined by personal surgeon preference and experience. Pre-operative antibiotics were administered in all cases. All IMNs used were long (300–460 mm) intramedullary titanium trochanteric fixation nails (Synthes) with proximal lag and distal locking screws. In those patients receiving a DHS, a dynamic hip screw system (Synthes) with a standard 38 mm, 135° barrel was used along with a dynamic compression plate containing between 5 and 10 holes through which 4.5-mm cortical screws were inserted. All procedures were performed on a traction table under intra-operative fluoroscopic guidance. Post-operatively, all patients received thromboprophylaxis and antibiotic treatment as per department protocol. Full blood count and urea and electrolyte blood tests were performed, and patients received regular physiotherapy and orthogeriatric review. Sutures or clips were removed 14 days post-operatively.

Further comparative data collected included the length of operation and inpatient stay, pre-/post-operative abbreviated mental test score (AMTS), 30-day mortality, 1-year mortality, dose area product (DAP) of the intra-operative fluoroscopy, complications and re-operations.

Pre- and post-operative haemoglobin (Hb) concentrations were also collected with the aim of calculating blood loss during the surgical procedure using the Haemoglobin dilution method, as described by Good et al. [16]. This method previously used by other studies for orthopaedic procedures is based on the premise that following a period of blood loss, fluid moves from the extravascular tissues into the blood vessels in order to restore blood volume, thus lowering Hb [16, 17]. The difference between pre- and post-operative Hb can then give an indication of the degree of blood loss that has occurred. The equation used assumes that the patient is euvolemic during the point at which pre- and post-op haemoglobin measurements are taken. Authors disagree about the time it takes for enough fluid to replace any blood lost and as such there is no standard time point used to determine post-op haemoglobin concentration. Previous research has used haemoglobin concentration measurements taken between the 3rd and 4th day after surgery [18]. This study aimed to use haemoglobin concentration on the fourth day after surgery; however, as patients did not have daily haemoglobin concentration measurements, this was not always the case. As such, if the haemoglobin concentration on the fourth day after surgery was not available, the next nearest measurement was used.

The equation used requires an estimation of the patient’s blood volume which was calculated using Nadler’s equation [19]. For two patients Nadler’s equation could not be used as weight/height data were not available and so average blood volume values for their respective sex were used as based on Enson et al. [20]. For three patients, weight data were available, but height/BMI data could not be found and so Gilcher’s rule of five was used where weight was multiplied by an expected blood volume per kg of body mass.

Statistical analysis was performed using IBM SPSS statistics 25. Welch’s *t*-test was used to compare mean values for operation length, DAP, blood loss and hospital stay between the two groups. Welch’s *t*-test was chosen before analysis of normality and homogeneity of variance due to the large difference in patient number in each treatment group. Analysis of normality using the Kolmogorov–Smirnov test and visual inspection of normal Q-Q plots revealed all data were normally distributed except for the length of stay values for the IMN group. Due to the extent of the non-normality in this group, the nonparametric Mann–Whitney *U* test was instead chosen to analyse length of stay data. Fisher’s exact test was used to test for significant differences between the groups with respect to categorical variables such as number of patients requiring blood transfusion and discharge destinations. Statistical significance was defined as *P* < 0.05.

## Results

A total of 1808 patients presented to our centre with a neck of femur fracture during the study period. Out of these patients, 86 suffered a subtrochanteric fracture of the femur, with 74 patients receiving an IMN and 12 receiving a DHS. Mean age was 84 (standard deviation: 8.89, range: 62–96) in the IMN group and 85 (standard deviation: 12.01, range: 64–99) in the DHS group. Gender distribution was 81% female and 19% male in the IMN group compared to 75% female and 25% male in the DHS group. In both groups, the majority of patients were admitted from home and received surgery within 36 h of admission (Table 1). The proportion of stable and unstable fractures was similar in both groups.

**Table 1:** Pre-operative characteristics of patients in both treatment groups

Descriptors	Intramedullary nail	Sliding hip screw
	Total *N* = 74 (%)	Total *N* = 12 (%)
Male, *n*	14 (18.92)	3 (25.00)
Female, *n*	60 (81.08)	9 (75.00)
Median age, years	84	85
Right-sided fracture, *n*	33 (44.59)	6 (50.00)
Left-sided fracture, *n*	41 (55.41)	6 (50.00)
*Seinsheimer classification*		
1	2 (2.70)	0 (0.00)
2A	4 (5.41)	0 (0.00)
2B	28 (37.84)	3 (25.00)
2C	7 (9.46)	3 (25.00)
3A	16 (21.62)	4 (33.33)
3B	6 (8.12)	0 (0.00)
4	4 (5.41)	1 (8.33)
5	7 (9.46)	1 (8.33)
Total stable	41 (55.41)	6 (50.00)
Total unstable	33 (44.59)	6 (50.00)
Pre-operative ASA (mean)	2.96	3.17
*Unknown*	6 (8.11)	1 (8.33)
1	1 (1.35)	0 (0)
2	15 (20.27)	1 (8.33)
3	38 (51.35)	7 (58.33)
4	14 (18.92)	3 (25.00)
Mean pre-operative AMTS	7.47	7.64
Mean post-operative AMTS	7.17	6.5
Body Mass Index	24.25	26.02
*Residence*		
Own home	66 (89.19)	10 (83.33)
Nursing care	3 (4.05)	1 (8.33)
Residential care	5 (6.76)	1 (8.33)
*Treatment delay*		
< 36 h	61(82.43)	11 (91.66)
> 36 h	13 (17.57)	1 (8.33)

The mean operative time in the IMN group was 106 min (95% CI: 97.76–114.60) compared to 100 min (95% CI: 70.95–129.71) in the DHS group (Table 2). This difference was not statistically significant (*P* = 0.683). The mean DAP was significantly higher (*P* = 0.000184) in the IMN group with a mean value of 288.9 mGy cm^2^ (95% CI: 236.26–341.47) compared to 150.3 mGy cm^2^ (95% CI: 102.07–298.53) in the DHS group. There was also a significant difference in the mean blood loss between the two groups, with the IMN group showing a significantly higher (*P* = 0.042) mean blood loss of 1029 ml (95% CI: 909.42–1148.07) compared to 776.ml (95% CI: 540.69–995.90) in the DHS group. Of those patients treated with an IMN, 54% required a blood transfusion in the five days following their operation. This figure was only 33% for those receiving a DHS; however, the difference was not statistically significant (*P* = 0.223). Furthermore, 10.8% of patients in the IMN group required a blood transfusion of greater than 2 units in the first five days following their operation. Whilst this was higher than the equivalent value for the DHS (0%), the difference did not show statistical significance (*P* = 0.593).

**Table 2:** Peri-operative outcome data for the two treatment groups

Descriptors	Intramedullary nail (95% CI)	Dynamic hip screw (95% CI)	*P* value
Mean operation time (minutes)	106.18 (97.76–114.60)	100.33 (70.95–129.71)	0.683
Mean DAP (mGy.cm^2^)	288.86 (236.26–341.47)	150.30 (102.07–298.53)	0.000
Mean blood loss (ml)	1028.74 (909.42–1148.07)	776.19 (540.69–995.90)	0.042
Mean total stay (days)	18.95 (15.58–22.30)	12.08 (8.52–15.65)	0.128
*Re-operation (%)*			
Implant failure	4 (5.41)	1 (8.33)	0.538
Infection	2 (2.70)	0 (0)	1.00
Sub-optimal fixation	1 (1.35)	0 (0)	1.00
Total	7 (9.46)	1 (8.33)	1.00
*Reduction*			
Open	54 (72.97)	9 (75.00)	1.00
Closed	20 (27.03)	3 (25.00)	1.00
Number requiring blood transfusion (%)	40 (54.05)	4 (33.33)	0.223
Number requiring blood transfusion > 2 Units (%)	8 (10.81)	0 (0)	0.593
*Discharged to (%)*			
Own home	32 (43.24)	3 (25.00)	0.345
Nursing care	6 (8.11)	1 (8.33)	1.00
Rehabilitation unit	28 (37.84)	5 (41.67)	1.00
Residential care	5 (6.76)	1 (8.33)	1.00
Died at hospital	3 (4.05)	1 (8.33)	0.458
*Mortality (%)*			
30 days post-op	3 (4.05)	2 (16.67)	0.141
1 year post-op	18 (24.32)	3 (25.00)	1.00

Values for number of patients requiring any blood transfusion and blood transfusion > 2 units only include patients who received these transfusions in the five days immediately following surgery

In the IMN group, a total of seven patients required re-operation. Deep infection requiring washout occurred in two patients, whilst implant failure due to proximal screw cut out occurred in four. A further patient required re-operation due to initial sub-optimal fixation and positioning of the nail. One case of implant failure on the background of a peri-prosthetic fracture accounts for the single re-operation in the DHS group. None of the differences in re-operation and complication rates between the two groups were found to be statistically significant (Table 2). No statistically significant differences were found in the number of patients receiving an open or closed reduction between the two groups. Reduction of the fracture in all 86 patients was deemed to be adequate.

The DHS group showed a trend towards shorter mean hospital stay of 12 days (95% CI: 8.52–15.65) compared to patients in the IMN group with a mean stay length of 19 days (95% CI: 15.58–22.30); however, this difference was not statistically significant (*P* = 0.128). There were differences in the discharge destination of patients between the two treatment groups as shown (Table 2); however, none of these differences showed any statistical significance.

In the IMN group, 3 patients (4%) died in the first 30 days following their operation compared to 2 (17%) in the DHS group. This difference was not statistically significant (*P* = 0.141). On the other hand, the IMN group showed a higher 1-year mortality with 18 patients (24%) dying within a year after their operation compared to 3 in the DHS group (25%). Again, this difference was not shown to be statistically significant (*P* = 1.00).

## Discussion

The treatment of subtrochanteric hip fractures is complex and technically challenging. This is partly due to the unique biomechanical and anatomical characteristics of the subtrochanteric area. The main structural support to this region is the calcar—a posteromedial bone element beginning distal to the lesser trochanter, travelling to the posteroinferior femoral neck. Koch found that this region can experience up to 1200 N of force in a 200 lb man when standing [21]. Furthermore, the subtrochanteric region experiences high stress levels due to the insertion of the hip abductors, iliopsoas tendon and short external rotators in the region. This along with the region’s poor vascular supply due to the extensive thick cortical bone makes the successful management of subtrochanteric fractures extremely difficult [22].

We found that at our centre, there was general adherence to UK national guidelines, with 74 subtrochanteric fractures (86%) being treated with an IMN. Patients in both treatment groups were similar in terms of demographic and pre-operative characteristics such as age, BMI, ASA grade and AMTS score. Interestingly, the majority of patients in both groups were female (81% In IMN group, 75% in DHS group). This is in agreement with previous work which have shown up to a 33% higher incidence of subtrochanteric fractures in females compared to males [23]. The reason for this increased incidence in females is unknown and could serve as a potential area worthy of further research.

### Blood loss

Studies have shown large amounts of hidden blood loss during intramedullary nailing, DHS insertion and other orthopaedic surgeries [17, 18]. As a result, when calculating blood loss, it was important to use a formula that estimated total blood loss rather than simply measuring volume of blood collected during surgery. A study by Gao et al. details four different methods for calculating surgical blood loss during a total knee arthroplasty, suggesting that the haemoglobin dilution method is the most accurate and so this was chosen [24].

Blood loss estimation using the above method showed a significantly lower mean blood loss for the DHS group (776 ml) compared to the IMN group (1029 ml). This is of particular importance in treatment of subtrochanteric fractures as the majority of patients are elderly with the average age in the studied cohort being 83 years. Anaemia has been identified as a risk factor for both morbidity and mortality in this elderly group and as such this significantly lower blood loss in the DHS group is of clinical benefit [25]. Furthermore, only 33% of those treated with a DHS required a blood transfusion in the five days following their operation, compared to 54% in those treated with an IMN. Although this difference was not found to be statistically significant (*P* < 0.05), it is worthwhile to note as another potential benefit of DHS use, particularly as blood transfusions have been associated with an increased risk of complications such as infection after surgery [26].

### Radiation exposure

Our results agree with a similar study whereby mean DAP was found to be significantly lower for patients treated with a DHS compared to an IMN [27]. The use of fluoroscopic image intensifiers in orthopaedics and the known pathological effects of radiation exposure continue to raise concerns as to their safety for both the patient and theatre staff [28]. To this end, DAP values were collected in order to assess the radiation risk patients endured. It represents the absorbed dose multiplied by the body area irradiated and so is a better indicator of overall risk than total radiation dose. Therefore, a potential advantage of using a DHS over an IMN to treat subtrochanteric fractures is a reduced risk of the pathological effects of exposure to ionising radiation. However, Malek et al. found that the risk of pathological radiation injury due to fluoroscopy in lower limb trauma operations is small and so the benefit of DHS in terms of radiation exposure may be of limited clinical benefit [28]. Nevertheless, according to Ionising Radiation (Medical Exposure) Regulations 2017 Guidelines patient exposure to radiation should be minimised wherever possible and using a DHS may help to do so [29].

All other outcome measures collected show no statistically significant difference between either of the two treatment groups. This may, in part, be due to the very small number of patients in the DHS group. Although this is understandable as current guidelines favour the use of an IMN, a larger DHS group may have revealed more statistical significance in the results. Another limitation of this study is the retrospective nature of the data collection and the lack of patient-reported outcome measures.

Current national guidelines recommend the use of IMN for the management of all subtrochanteric fractures. Upon further inspection of the guidelines, it becomes clear that this is due to concerns with reportedly high rates of non-union following treatment of subtrochanteric fractures with a DHS. This conclusion is based on two studies investigating the use of IMN’s compared to DHS in subtrochanteric fractures. The first by Ekstrom et al. finds that only 1/13 patients treated by DHS showed non-union, whilst 0/19 of those treated with an IMN showed non-union [30]. The second study conducted by Rahme et al. found that 8/29 treated with a DHS showed non-union, whilst this figure was 0/29 for those treated with an IMN [31]. The guidelines are currently based on only two studies with relatively small samples sizes and one of which shows a very minor difference in non-union, even concluding that there were no differences in the functional outcome and complication rates between the two treatment groups and even that the patients treated with a DHS showed a lower re-operation rate [30]. Unfortunately, due to department policy, there is a lack of routine follow-up for patient’s presenting with neck of femur fractures at our centre and so it was not possible to assess fracture union in our retrospective study. However, patients who had specific problems/concerns following surgery were followed up, with none of these patients showing any evidence of non-union. Previous studies have also shown no significant difference in union between IMN and DHS patients and we would suggest that these differing results in the literature make it difficult to draw a definitive conclusion about differences in union rates between the two implants [10, 30]. Furthermore, both groups showed similar results in terms of post-operative complications, with no significant differences found in total rate of re-operation and implant failure.

Cost analysis by the UK National Institute for Health and Care Excellence (NICE) reveals a large difference in the cost of the two implants, with the average price of a DHS implant being £252.51 compared to an average of £1,175.50 for a long IMN [2]. Of course, differences in cost should not play a part in clinical decision making should there be any significant clinical difference in favour of the use of a particular implant. However, the results of this study fail to show any such significant differences in favour of use of the IMN and in fact indicate that DHS may perform better in terms of important variables such as blood loss and radiation exposure. The DHS group showed a trend towards having a lower mean length of hospital stay than the IMN group. Although this difference was not shown to be significant in our cohort, it is worthy of note as a shorter hospital stay combined with reduced cost of the implant itself is a major economic advantage of treatment with a DHS.

One key problem with the treatment of subtrochanteric fractures that might contribute to the difference of opinions in the literature is the lack of a consistently used, universally accepted classification system. Three systems are currently commonly used: the AO/OTA system, the Russell-Taylor System and the Seinsheimer system which makes it hard to compare the results of different studies and put into place recommended guidelines for different subtypes of subtrochanteric fractures. Developing a universally used system could prove to be key in improving treatment as biomechanical studies have shown that dynamic hip screws and intramedullary nails have differing stability when used in different subtypes of subtrochanteric fractures. Wang et al. found that the DHS needed integrity of the medial subtrochanteric cortex as in stable Seinsheimer fracture types I and II in order to provide stable fixation [15]. In these situations, the DHS lateral plate was able to act as a ‘tension band’ allowing tension stress to be transmitted to the intact medial cortex. However, in situations where the medial subtrochanteric cortex is not intact as in unstable Seinsheimer type III and IV fractures, this stress is transferred to the lateral side plate of the DHS leading to bending of the plate and high compression stress on the lateral cortex of the femur and subsequent implant failure. It was determined that in such situations, IMNs biomechanically perform much better and are able to provide stable fixation as stress can be transmitted through the nail to the distal femur rather than the weak subtrochanteric cortex. However, these data are yet to be tested in a clinical setting and further research should be conducted into determining the suitability of both implants in different subtrochanteric fracture subtypes.

It must be acknowledged that this study is not without limitations. For example, the number of patients treated with a DHS is small, which may lead to a lack of statistical power. However, the studies currently used to inform UK national subtrochanteric fracture management guidelines suffer from a similar problem, with only 13 and 29 patients in the DHS group, respectively. Furthermore, our study cohort includes 74 patients treated with an IMN, compared to the smaller numbers of 19 and 29 seen in these previous studies. The retrospective nature of this study, together with the lack of regular follow-up, as is standard practice in the UK for this patient cohort, means we were not able to collect further follow-up data such as fracture union and time to full weight bearing, or set clear a-priori criteria for situations in which an IMN or DHS should be used. Due to the above limitations, we cannot draw any firm conclusions form this study; however, it is important to challenge existing practices to improve patient outcomes in a continuously challenging economic environment for healthcare. The current UK guidelines on treatment of subtrochanteric fractures may be too rigid and based on limited evidence.

## Conclusion

Although current national guidelines in the UK recommend treating all subtrochanteric fractures with an IMN, the outcome measures we assessed in our study did not demonstrate that treating these fractures with an IMN was superior to using a DHS. Furthermore, the DHS group showed a lower estimated blood loss and a reduced DAP. This, along with the reduced financial cost associated with DHS, may support the use of DHS over IMN for certain subtrochanteric fractures of the femur. There may not be a single favourable implant for the treatment of subtrochanteric fractures as a whole; instead, different subtypes of fracture may be amenable to the use of a different fixation device. Adoption of a universal fracture classification system for subtrochanteric fractures may aid in the development of specific guidelines for these different fracture subtypes and the optimum fixation construct. With an ageing population during a period of increasing financial restraints on a national health service, our national guidelines for fracture fixation should evolve with emerging evidence and provide comprehensive guidance to shape surgical practice.
